# Body weight perception, disordered weight control behaviors, and depressive symptoms among Korean adults: The Korea National Health and Nutrition Examination Survey 2014

**DOI:** 10.1371/journal.pone.0198841

**Published:** 2018-06-14

**Authors:** Yongjoo Kim, S. Bryn Austin, S. V. Subramanian, Ichiro Kawachi

**Affiliations:** 1 Department of Social and Behavioral Sciences, Harvard T.H. Chan School of Public Health, Boston, Massachusetts, United States of America; 2 Division of Adolescent and Young Adult Medicine, Boston Children’s Hospital, Boston, Massachusetts, United States of America; Hong Kong Polytechnic University, HONG KONG

## Abstract

**Background/ Purpose:**

Despite emerging evidence suggesting harmful influences of accurate weight perception on psychological health among individuals with obesity, little is known about the association in Asian populations. The aim of this study was investigate the association between body weight perception and depressive symptoms among Korean adults, and potential differential associations across gender.

**Methods:**

We used data from the sixth Korea National Health and Nutrition Examination Survey in 2014, comprising 3,318 female (n = 1,876) and male (n = 1,442) participants, aged 19–65 years, with no history of depression and a body-mass index (BMI)> = 18.5kg/m^2^. Depressive symptoms were measured by the Patient Health Questionnaire-9 Korean version. Weight perception patterns were categorized by comparing self-perceived and objectively measured weight status. Gender-stratified four-level multilevel linear models adjusted for age, BMI, menopause, education, income, marital status, urbanicity, chronic conditions, exercise, smoking, and alcohol use. Subgroup analyses were performed across BMI category.

**Results:**

Among women with obesity, those who underperceived their weight status reported fewer depressive symptoms compared to those who accurately perceived their weight status (β = -1.25, p<0.05). Among women with normal weight, those who overperceived their weight status reported more depressive symptoms compared to those who accurately perceived their weight status (β = 1.00, p<0.05). The same associations were not found in men.

**Conclusion:**

Awareness-oriented strategies for obesity prevention and weight management focused on providing information on weight status may need to consider unintended consequences of accurate weight perception on mental health among individuals with obesity, particularly among women.

## Introduction

The global obesity epidemic has placed obesity prevention and reduction among the most urgent public health issues [1]. Previous studies have reported that weight misperception among individuals with overweight/obesity might hinder the successful implementation of obesity prevention and management interventions [2–4], and thus have advocated the use of “awareness-raising” approaches in public health and clinical practice [2–6]. Overall, awareness-based strategies seek to help individuals with overweight/obesity to correctly recognize their weight status, intending to encourage self-oriented weight management and compliance with treatments [5–8]. School-based body-mass index (BMI) screening followed by BMI “report cards” sent to parents is one example of an awareness-oriented strategy employed in obesity prevention for children and adolescents [5, 7–10]. Clinicians in primary care settings are also trained to evaluate patients’ BMI and provide accurate information on their weight status with appropriate behavioral modification and treatments if necessary [3, 5–8].

In South Korea, the prevalence of obesity has increased 1.4-fold between 1998 and 2007 among Korean men (from 26.0% to 36.3%), but remained stable among Korean women (from 26.5% to 27.6%) [11]. Prior Korean studies reported high prevalences of weight misperception among Korean adults [11, 12] and adolescents [13, 14] and emphasized the need for public health and clinical strategies that can correct weight misperception among individuals with obesity [11–13]. One of the fundamental premises of this perspective is that individuals who correctly identify their health risk (i.e., overweight status) would be more likely to initiate appropriate remedial steps including physical activity and a healthy diet [2–5, 7–9, 13, 15]. This perspective has been supported by some cross-sectional studies showing that weight misperception among adolescents [3, 13] and adults [2] with overweight/obesity was inversely associated with their intentions/attempts to control weight.

However, recent prospective and cross-sectional studies have raised concerns about these awareness-based approaches in the context of obesity prevention/management as they could lead to unintended consequences stemming from weight stigma [5, 7–9, 15, 16]. Some studies have found that accurate weight perception was associated with increased weight gain [7, 17], higher blood pressure [9], depressive symptoms [15, 16], and disordered weight control behaviors (DWCB, including fasting, skipping meals, self-induced vomiting, and laxative/diuretic/diet pill use) [18] among adolescents and young adults with overweight/obesity. Together with recent findings linking self-perception of obeisty with experiences of being bulllied [19, 20], these studies suggested that accurate perception of overweight/obese status can either be directly associated with internalizing weight stigma or amplify the negative impacts of stigma that those individuals encounter due to their body size, particularly in contexts where fat bias is prevalent [5, 7–10, 15–18].

Recent studies have shown that weight stigma is pervasive in South Korea. A recent study carried out in 72 countries found that Koreans have the strongest preference for thin individuals over individuals with overweight/obesity, compared to participants from all other countries [21]. Traditional Korean society associated a moderately “plump body” with positive values (e.g., beauty and wealth), but a “thin body” with negative images (e.g., illness and poverty) [12, 22]. However, the rapid social changes characterized by industrialization, urbanization, and Westernization over the past half-century in South Korea have flipped the traditional norms and values regarding body image [12, 23, 24]. As a result, the thin-ideal is prevalent in South Korea, and having a slim body is viewed as an essential component for a successful marriage and career, particularly among Korean women [23].

However, despite this society-wide obsession with the thin-ideal, little is known about the potential harmful associations between accurate weight perception and unfavorable health outcomes among individuals with obesity in South Korea. More broadly, among Asian countries, there have been few studies examining such associations. Therefore, to fill the gap in the current knowledge, by using a nationally representative dataset of Koreans, we investigated whether weight perception was associated with depressive symptoms among Korean adults aged 19–65 years with no prior history of depression. Previous studies have shown that perception of being normal weight (vs. perception of being obese) is associated with more favorable psychological outcomes such as decreased depressive symptoms [15, 16, 25, 26]. Therefore, we hypothesized that weight status underpreception among individuals with obesity would be protectively associated with depressive symptoms, whereas weight status overperception among individuals with normal weight would be positively associated with depressive symptoms. Given the prior evidence showing that the thin-ideal and body dissatisfaction have disproportionately affected more women than men [23, 27], we further hypothesized that the associations would be stronger among women than men.

## Methods

### Study population

We used the Sixth Korea National Health and Nutrition Examination Survey (KNHANES VI-2) 2014, an ongoing nationwide surveillance system administered by the Korea Centers for Disease Control and Prevention (K-CDC) [28]. Through its health interviews, examinations and nutrition surveys, KNHANES collects information on the sociodemographic, behavioral, and clinical profiles of Koreans [28]. The survey uses stratified multistage clustered area-probability sampling to draw a nationally representative sample of non-institutionalized Koreans [28] with towns/neighborhoods (e.g., Eup/Myeon/Dong) as primary sampling units (PSU), households within each PSU as secondary sampling units (SSU), and all individuals aged 1 year or older within each SSU as final sampling units.

**Fig 1:**
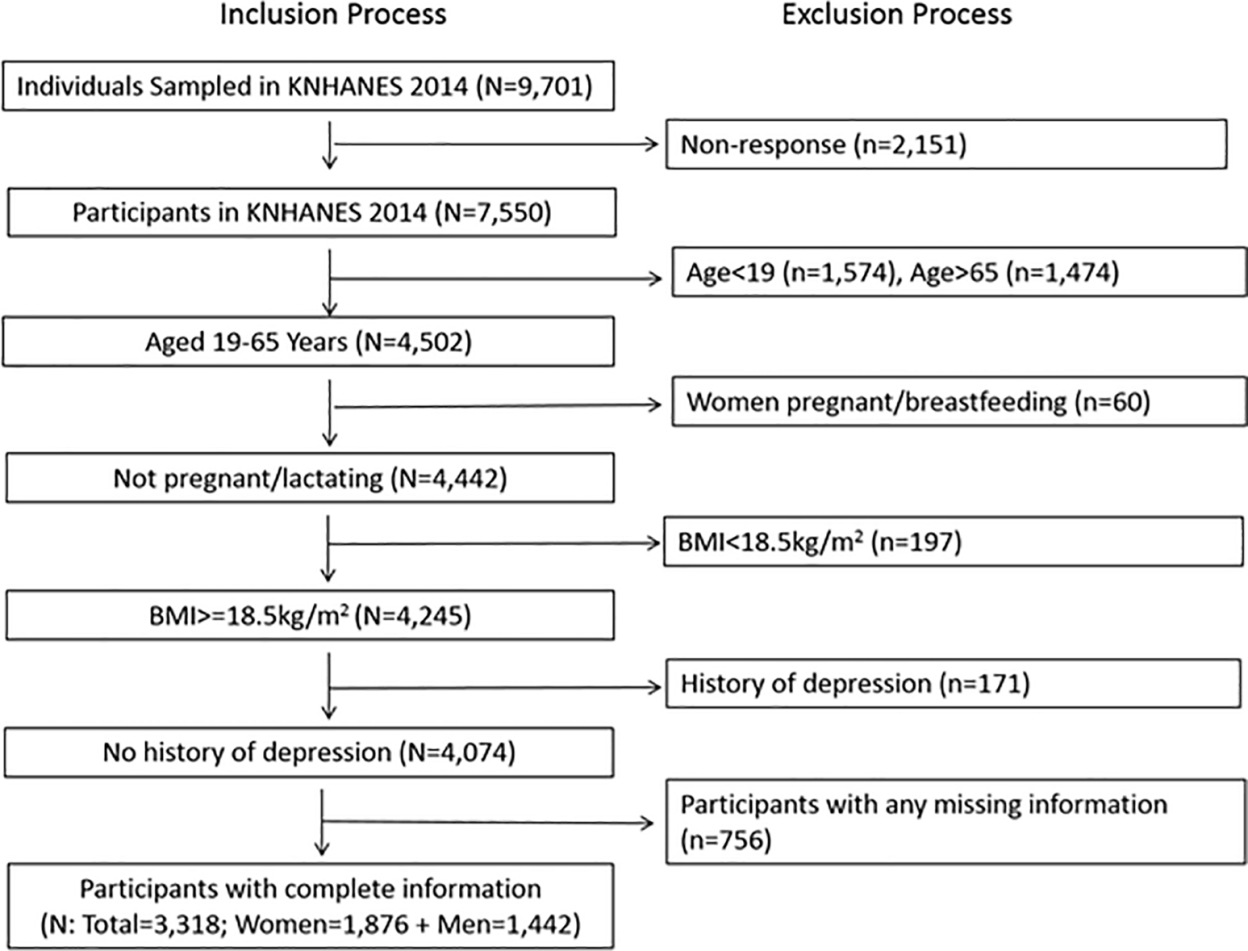
Inclusion and exclusion processes for the analytic sample from the Korea National Health and Nutrition Examination Survey (KNHANES) in 2014.

Among the initially targeted individuals, 77.8% responded to the survey (Fig 1). Of these, we excluded individuals aged less than 19 years (n = 1,574), since the depression screening was only administered to those aged 19 years or older. We further excluded participants who were older than 65 years (n = 1,474), pregnant or breastfeeding (n = 60), underweight (BMI<18.5kg/m^2^, n = 197); and reported a history of physician-diagnosed depression (n = 171). Among the 4,074 participants who met these inclusion criteria, we excluded those who had missing information on any variables used in our analysis (n = 756), resulting in a total of 3,318 participants (1,876 women and 1,442 men) in the analytic sample (81.4% of those who met the inclusion criteria and 43.9% of the entire participants of KNHANES VI-2). The survey was conducted with the informed consent of the participants. Since our analytical data did not involve personal identifiers, the Office of Human Research Administration of the Harvard T.H. Chan School of Public Health exempted this study from IRB review.

### Measures

#### Depressive symptoms

Depressive symptoms were measured by the Patient Health Questionnaire-9 Korean version (PHQ-9K) through face-to-face interviews by trained staff members in a mobile examination center. The Patient Health Questionnaire-9 (PHQ-9) is a well-validated, widely-used screening instrument for depression, consisting of a 9-item questionnaire on depression symptomatology during the previous two weeks [29]. Each item includes a 4-point scale response option, including “3: nearly every day,” “2: more than half of the days,” “1: several days,” and “0: not at all,” with a total score ranging from 0 to 27 [29]. Previous studies suggested that PHQ-9K is an appropriate measure to screen depression with an internal consistency reliability of 0.88 and convergent validity of 0.74 among Korean adults [30–32].

#### Weight status perception pattern

Anthropometric inforamtion was obtained through the health examination at the moblie examination center perfomed by the trained staff of KNHANES. While participants were shoeless and wearing a light gown, height (by using a portable stadiometer to the nearest 1mm) and weight (by using an electronic scale to the nearest 100g) were measured [28]. After calculating body-mass index (BMI, kg/m^2^), we used the BMI value of 25.0kg/m^2^ as the cut-off to define obesity (BMI> = 25kg/m^2^) based on the revised Asia-Pacific BMI criteria by the World Health Organization [33, 34]. Self-perception of body weight was assessed with the question, “How would you describe your body shape?” with the response options: “very thin,” “slightly thin,” “normal,” “slightly obese,” and “very obese” [28]. The responses were regrouped into three categories: “very/slightly thin,” “normal,” and “slightly/very obese.” By comparing the perceived weight status with the measured weight status, we created a categorical variable for weight status perception pattern: accurate perception (perceived weight status = measured weight status), overperception (perceived weight status > measured weight status), and underperception (perceived weight status < measured weight status).

#### Disordered weight control behaviors

Weight control behaviors were measured by a two-stage questionnaire. First, all participants were asked whether they had attempted to control their weight during the previous 12 months with the response options: “to lose,” “to maintain,” “to gain,” and “no attempts.” Then, those who responded “to lose” or “to maintain” were asked whether they had used the following methods for weight control: (a) “fasting (> = 24 hours),” (b) “skipping meals,” (c) “one-food dieting (i.e., eating only one type of food for weight control, e.g., bananas, potatoes, milk),” and (d) “using unprescribed diet pills,” with binary (ever/never) response options for each behavior [28]. We created an indicator variable for each (a)-(d) behavior. Based on the definition of disordered weight control behaviors (DWCB) from the K-CDC [35] and prior literature [36], we generated a composite indicator variable for DWCB, indicating whether or not participants engaged in one or more of the (a)-(d) weight control behaviors during the previous 12 months.

#### Covariates

Menstrual status (for women) was grouped as having menstrual cycles and menopause. Marital status was classified as “married,” “never married,” and “married, but separated/divorced/widowed.” To assess economic status, equivalised total household income, or self-reported household total income over the square root of the number of family members, was grouped into quartiles. Based on the self-reported highest degree completed, educational attainment was categorized into three groups: < = middle school graduate, high school graduate, and > = college graduate.

Previous studies have found that perception of weight status is associated with physical activity [37], cigarette smoking [38–40] and alcohol drinking [14, 41]. Given the well-documented relationship between such behavioral factors and depression [42–47], we included smoking, alcohol drinking, and physical activity as covariates. High-risk alcohol use (yes/no) was defined as drinking seven or more glasses of alcohol for men and five or more for women per occasion, on two or more occasions every week over the past year. Smoking status was grouped as current smokers (> = 5 packs over lifetime and having smoked over the past month), past smokers (> = 5 packs over lifetime but not having smoked over the past month), and non-smokers (<5 packs over lifetime). The Korean version of the Global Physical Activity Questionnaire was used to assess moderate-to-vigorous leisure-time physical activity (LTPA), defined as having one or more occasions of either moderate LTPA (i.e., having at least 10 minutes of sports/exercise/recreation activities that slightly increased heart rate or breathing) or vigorous LTPA (i.e., having at least 10 minutes of sports/exercise/recreation activities that substantially increased heart rate or breathing) during the previous week. Based on the self-reported history of physician-diagnosed chronic diseases, having a history of one or more severe chronic conditions such as coronary heart disease, stroke, and cancer was identified and included as an indicator variable.

### Statistical analyses

To investigate whether weight perception was cross-sectionally associated with depressive symptoms, we performed four-level multilevel random intercept linear models, in which individuals (level 1: N = 3,318) were nested in households (level: N = 2,057), nested in neighborhoods (level 3: N = 192), and nested in geographic areas (level 4: N = 16). We built three models in sequence as follows. Model 1 adjusted for BMI (kg/m^2^) and age (years) both as continuous variables, marital status, education, household income (quartile), urbanicity, severe chronic condition, smoking, high-risk alcohol use, and exercise. To understand the association between DWCB and depressive symptoms, as well as the extent to which DWCB explains the association between weight perception and depressive symptoms, Model 2 added overall DWCB based on Model 1. To investigate method-specific associations of DWCB with depressive symptoms, Model 3 added four indicator variables (fasting, skipping meals, one-food dieting, using unprescribed diet pills), instead of overall DWCB, building on Model 1.

We built the aforementioned models stratified by gender based on the marginally significant interaction between gender and weight perception pattern in our sample (p = 0.07), and further performed subgroup analysis across BMI category (normal and obese). Additionally, given the rapid change in social norms regarding body image in South Korea over the past decades [12, 23, 24], we performed stratified analysis across younger (i.e., aged 19–40 years) and older (i.e., aged 41–65 years) aged groups. For all multilevel modeling, we used MLwiN 3.01 (Center for Multilevel Modeling, Bristol University) by adapting a Markov Chain Monte Carlo (MCMC) with Metropolis-Hastings algorithm with uninformative priors, a Bayesian approach implemented in MLwiN, and set the level of significance at 0.05 (two-sided) [48].

While obesity is defined as a BMI value of 30.0 (kg/m^2^) or above in Western populations, the Korean Society for the Study of Obesity has suggested 25.0 as the cut-off to define obesity in Koreans, based on the revised Asia-Pacific BMI criteria issued by the World Health Organization [33, 34]. However, not ever individual knows their exact weight and hence, there could be random misclassification of weight status just above or below the BMI threshold of 25.0 kg/m^2^, which could bias/attenuate the true association. Conversely, as an individual’s BMI moves further away from the cut-off value (i.e., 25.0kg/m^2^), such random misclassification becomes less likely. We performed sensitivity analyses by looking at the association among subsets of participants whose BMI fall more squarely within the range of normal weight or obesity, where random misclassification of weight status is less likely. Thus, we imposed some hypothetical distances from the BMI cut-off for obesity (e.g., by 1.0, 1.5, and 2.0 units) and re-ran the aforementioned models (Models 1–3) among individuals within the alternative ranges of normal weight (18.5< = BMI<24.0kg/m^2^, <23.5, and <23.0) as well as obesity (BMI> = 26.0kg/m^2^, > = 26.5, and > = 27.0).

In order to test whether our multilevel linear MCMC modeling approach is robust or sensitive to the non-normal distribution of the outcome, PHQ-9K scores, we also used a multilevel binomial MCMC modeling strategy with a cut-off of 5 (or above) to define depression, based on previous literature among Koreans [30]. Additionally, to address missing data in 18.6% of our study population, we performed another sensitivity analysis by using a multiple imputation analysis strategy implemented in Stata/MP 15.0 with the chained equation method by creating 20 imputed datasets after 100 iterations for the burn-in period [49].

## Results

### Descriptive statistics

Among our sample, women (compared to men) had on average higher depressive symptom scores, a higher prevalence of DWCB and weight status overperception, but a lower prevalence of weight status underperception (Table 1).

**Table 1:** Descriptive characteristics of the study sample (N = 3,318): Korean adults aged 19–65 years with BMI> = 18.5kg/m^2^ and no history of depression.

		Women (N = 1,876)	Men (N = 1,442)	
		Mean (SE)^a^	Mean (SE) ^a^	p^b^
PHQ-9K		2.9 (0.1)	2.0 (0.1)	<0.01
Age	(years)	42.5 (0.4)	41.3 (0.4)	0.02
BMI	(kg/m^2^)	23.2 (0.1)	24.5 (0.1)	<0.01
		N (%)^a^	N (%)^a^	p^b^
Weight perception	Underperception	156 (7.7)	347 (23.2)	<0.01
	Overperception	518 (28.4)	132 (9.5)	
DWCB	Overall	257 (15.0)	115 (8.4)	<0.01
	Fasting	52 (3.4)	35 (2.6)	0.27
	Skipping meals	205 (12.1)	92 (6.8)	<0.01
	One-food diet	38 (2.3)	10 (0.9)	0.01
	Unprescribed diet pills use	21 (1.2)	2 (0.1)	0.02
Menopause	Yes	744 (32.9)	-	
Marital status	Not married	299 (20.0)	332 (30.2)	<0.01
	Married, but separated	165 (7.2)	59 (3.7)	
Education	< = Middle school	469 (20.7)	256 (14.3)	<0.01
	High school	724 (41.3)	556 (41.9)	
Household income	1st	177 (9.4)	103 (6.3)	<0.01
	2nd	491 (25.8)	333 (23.5)	
	3rd	615 (32.8)	511 (35.3)	
Urbanicity	Rural	267 (13.4)	227 (14.8)	0.26
Severe chronic conditions^c^	Yes (> = 1)	116 (5.4)	71 (3.8)	0.04
Physical activity^d^	Yes (> = 1day/week)	611 (33.6)	665 (47.4)	<0.01
Smoking	Current smoker	87 (4.9)	643 (44.6)	<0.01
	Past smoker	60 (3.4)	414 (25.6)	
High-risk drinking^e^	Yes	119 (6.8)	327 (22.9)	<0.01

Data source: the Sixth Korea National Health and Nutrition Examination Survey in 2014

Abbreviations: PHQ-9K (Patient Health Questionnaire-9 Korean version), BMI (body-mass index), DWCB (disordered weight control behavior)

a) Survey weighted means and standard errors for continuous variables, and raw frequencies and survey weighted column percentages for binary/categorical variables were presented.

b) P-value was based on the test of difference in means for continuous variables and proportions for binary/categorical variables between women and men.

c) Severe chronic conditions included history of stroke, coronary heart disease, and cancer.

d) Physical activity indicates having at least 1day/week of moderate-to-rigorous leisure time physical activity

e) High-risk drinking indicates habitual alcohol drinking episodes (> = 2/week over the past year) with > = 7 glasses for men, or > = 5 glasses for women, per each episode.

### Multilevel analysis

Overall, weight perception was associated with depressive symptoms among women, but not among men. Compared with accurate perception, weight status underperception was inversely associated with depressive symptoms among women with obesity (*β* = -1.25, 95% CI: -2.44, -0.01), suggesting a protective relationship of weight status underperception with depression among those with obesity (Table 2). The same association was not found among men (Table 3). However, among women with normal weight, weight status overperception was positively associated with depressive symptoms (*β* = 1.00, 95% CI: 0.54, 1.45), suggesting a harmful relationship of weight status overperception with depression among those with normal weight. Again, no association was found among men. The differential associations across women and men were confirmed by significant interaction terms for gender and weight perception (Tables A and B in S1 File). The associations between weight perception and depressive symptoms remained consistent before and after adjusting for DWCB (both overall and method-specific).

**Table 2:** Association of weight status perception pattern and disordered weight control behaviors with depressive symptoms (PHQ-9K) among Korean women (N = 1,876) aged 19–65 years with BMI> = 18.5kg/m^2^ and no history of depression.

	Beta (95% CI)		
	Model 1^a^^b^	Model 2^a^^c^	Model 3^a^^d^
*All Women* (N = 1,876)			
Underperception	0.19 (-0.40, 0.76)	0.19 (-0.39, 0.76)	0.19 (-0.38, 0.77)
Overperception	0.45* (0.10, 0.79)	0.39* (0.05, 0.74)	0.39* (0.05, 0.75)
DWCB		0.88* (0.41, 1.35)	
Fasting			1.90* (0.93, 2.85)
Skipping meals			0.64* (0.12, 1.15)
One-food diet			0.24 (-0.92, 1.32)
Unprescribed diet pills use			-0.40 (-1.86, 1.10)
Women with Obesity (N = 466)			
Underperception	-1.25* (-2.44, -0.01)	-1.30* (-2.56, -0.05)	-1.40* (-2.66, -0.20)
DWCB		0.85 (-0.01, 1.72)	
Fasting			-0.24 (-1.83, 1.33)
Skipping meals			1.61* (0.58, 2.61)
One-food diet			0.08 (-1.86, 2.00)
Unprescribed diet pills use			-1.06 (-3.29, 1.19)
Women with Normal Weight (N = 1,410)			
Underperception	0.14 (-0.55, 0.84)	0.17 (-0.50, 0.83)	0.13 (-0.56, 0.81)
Overperception	1.00* (0.54, 1.45)	0.91* (0.48, 1.35)	0.93* (0.49, 1.38)
DWCB		0.85* (0.33, 1.39)	
Fasting			3.40* (2.15, 4.63)
Skipping meals			0.32 (-0.27, 0.93)
One-food diet			0.17 (-1.16, 1.51)
Unprescribed diet pills use			-0.93 (-2.86, 1.01)

Abbreviations: PHQ-9K (Patient Health Questionnaire-9 Korean version), DWCB (disordered weight control behavior), BMI (body-mass index)

a) All models were based on four-level random intercept model, in which individuals at level 1 were nested within households at level 2, nested within neighborhoods at level 3, and nested within wider geographic areas at level 4.

b) Model 1 included weight perception pattern (accurate perception as reference), BMI (kg/m^2^), age (years), menopause, education, marital status, household income, urbanicity, severe chronic conditions, moderate-to-rigorous leisure time physical activity, smoking, and high-risk alcohol drinking.

c) Model 2 added DWCB (yes versus no) based on Model 1.

d) Model 3 added four indicator variables such as fasting (yes vs. none), skipping meals (yes vs. none), one-food diet (yes vs. none), and unprescribed diet pills use (yes vs. none), instead of overall DWCB, based on Model 1, to investigate method-specific association.

“*” indicates p-value <0.05 (two-sided).

**Table 3:** Association of weight status perception pattern and disordered weight control behaviors with depressive symptoms (PHQ-9K) among Korean men (N = 1,442) aged 19–65 years with BMI> = 18.5kg/m^2^ and no history of depression.

	Beta (95% CI)		
	Model 1^a^^b^	Model 2^a^^c^	Model 3^a^^d^
*All Men* (N = 1,442)			
Underperception	0.14 (-0.25, 0.52)	0.13 (-0.25, 0.53)	0.15 (-0.25, 0.52)
Overperception	-0.09 (-0.62, 0.44)	-0.12 (-0.65, 0.41)	-0.18 (-0.70, 0.35)
DWCB		0.26 (-0.30, 0.83)	
Fasting			-0.45 (-1.48, 0.54)
Skipping meals			0.23 (-0.42, 0.88)
One-food diet			1.63 (-0.17, 3.39)
Unprescribed diet pills use			8.25* (4.28, 12.05)
Men with Obesity (N = 590)			
Underperception	0.01 (-0.62, 0.64)	-0.01 (-0.65, 0.61)	0.03 (-0.62, 0.67)
DWCB		-0.19 (-0.88, 0.49)	
Fasting			-0.66 (-1.86, 0.57)
Skipping meals			0.04 (-0.73, 0.80)
One-food diet			-1.39 (-4.41, 1.65)
Unprescribed diet pills use			5.36* (-0.08, 10.81)
Men with Normal Weight (N = 852)			
Underperception	0.30 (-0.25, 0.85)	0.31 (-0.23, 0.84)	0.32 (-0.23, 0.87)
Overperception	-0.21 (-0.78, 0.38)	-0.27 (-0.87, 0.34)	-0.30 (-0.90, 0.28)
DWCB		0.95* (0.01, 1.90)	
Fasting			-0.32 (-2.09, 1.47)
Skipping meals			0.22 (-0.95, 1.40)
One-food diet			2.85* (0.53, 5.11)
Unprescribed diet pills use			10.70* (4.78, 16.81)

Abbreviations: PHQ-9K (Patient Health Questionnaire-9 Korean version), DWCB (disordered weight control behavior), BMI (body-mass index)

a) All models were based on four-level random intercept model, in which individuals at level 1 were nested within households at level 2, nested within neighborhoods at level 3, and nested within wider geographic areas at level 4.

b) Model 1 included weight perception pattern (accurate perception as reference), BMI (kg/m^2^), age (years), education, marital status, household income, urbanicity, severe chronic conditions, moderate-to-rigorous leisure time physical activity, smoking, and high-risk alcohol drinking.

c) Model 2 added DWCB (yes versus no) based on Model 1.

d) Model 3 added four indicator variables such as fasting (yes vs. none), skipping meals (yes vs. none), one-food diet (yes vs. none), and unprescribed diet pills use (yes vs. none), instead of overall DWCB, based on Model 1, to investigate method-specific association.

“*” indicates p-value <0.05 (two-sided).

Overall DWCB was positively associated with depressive symptoms among women (*β* = 0.88, 95% CI: 0.41, 1.35), but not among men. In terms of method-specific associations, fasting (*β* = 1.90, 95% CI: 0.93, 2.85) and skipping meals (*β* = 0.64, 95% CI: 0.12, 1.15) were positively associated with depressive symptoms among women, but not among men. When further stratified by BMI category, one-food dieting (*β* = 2.85, 95% CI: 0.53, 5.11) was positively associated with depressive symptoms among men with normal weight, but not among other subgroups.

Our tests of interaction between age groups (19–40 vs. 41–65 years) and weight status perception and weight control behaviors (Tables C and D in S1 File) showed no evidence of statistically significant interaction across age groups. However, the magnitude of the association was more pronounced in the younger age group (19–40 years) than the older age group (41–65 years). As shown in Tables 4 and 5, weight status overperception (vs. accurate perception) among individuals aged 19–40 years with normal weight was associated with an average 1.04 point (95% CI: 0.49, 1.60) higher depressive symptom score, compared to 0.63 points (95% CI: 0.22, 1.05) among those aged 41–65 years with normal weight. Similarly, DWCB (yes vs. none) among individuals aged 19–40 years was associated with 1.00 point (95% CI: 0.48, 1.52) higher depressive symptom score, whereas the magnitude of the association was 0.69 point (95% CI: 0.20, 1.18) for those aged 41–65 years.

**Table 4:** Association of weight status perception pattern and disordered weight control behaviors with depressive symptoms (PHQ-9K) among Koreans aged 19–40 years with BMI> = 18.5kg/m^2^ and no history of depression.

	Beta (95% CI)		
	Model 1^a^^b^	Model 2^a^^c^	Model 3^a^^d^
*Aged 19–40 Years* (N = 1,307)			
Underperception	-0.37 (-0.98, 0.23)	-0.27 (-0.89, 0.32)	-0.32 (-0.91, 0.28)
Overperception	0.48* **(0.02, 0.94)**	0.37 (-0.10, 0.82)	0.35 (-0.10, 0.80)
DWCB		1.00* **(0.48, 1.52)**	
Fasting			1.53* **(0.53, 2.55)**
Skipping meals			0.67* **(0.07, 1.27)**
One-food diet			0.29 (-1.15, 1.72)
Unprescribed diet pills use			0.62 (-1.30, 2.57)
*Aged 19–40 years with normal weight*: (N = 942)			
Underperception	-0.67 (-1.35, 0.03)	-0.53 (-1.20, 0.12)	-0.59 (-1.27, 0.11)
Overperception	1.04* **(0.49, 1.60)**	0.83* **(0.26, 1.39)**	0.84* **(0.29, 1.39)**
DWCB		1.07* **(0.40, 1.73)**	
Fasting			2.94* **(1.57, 4.29)**
Skipping meals			0.34 (-0.39, 1.06)
One-food diet			0.33 (-1.22, 1.89)
Unprescribed diet pills use			0.37 (-1.98, 2.71)
*Aged 19–40 years with obesity*: (N = 365)			
Underperception	-0.71 (-2.02, 0.59)	-0.64 (-1.97, 0.76)	-0.60 (-1.93, 0.71)
DWCB		0.54* **(-0.33, 1.43)**	
Fasting			-0.50 (-2.04, 1.02)
Skipping meals			1.09* **(0.07, 2.06)**
One-food diet			0.35 (-2.83, 3.55)
Unprescribed diet pills use			-0.90 (-4.14, 2.38)

Abbreviations: PHQ-9K (Patient Health Questionnaire-9 Korean version), DWCB (disordered weight control behavior), BMI (body-mass index)

a) All models were based on four-level random intercept model, in which individuals at level 1 were nested within households at level 2, nested within neighborhoods at level 3, and nested within wider geographic areas at level 4.

b) Model 1 included weight perception pattern (accurate perception as reference), BMI (kg/m^2^), age (years), menopause (for women), education, marital status, household income, urbanicity, severe chronic conditions, moderate-to-rigorous leisure time physical activity, smoking, and high-risk alcohol drinking.

c) Model 2 added DWCB (yes versus no) based on Model 1.

d) Model 3 added four indicator variables such as fasting (yes vs. none), skipping meals (yes vs. none), one-food diet (yes vs. none), and unprescribed diet pills use (yes vs. none), instead of overall DWCB, based on Model 1, to investigate method-specific association.

“*” indicates p-value <0.05 (two-sided).

**Table 5:** Association of weight status perception pattern and disordered weight control behaviors with depressive symptoms (PHQ-9K) among Koreans aged 41–65 years with BMI> = 18.5kg/m^2^ and no history of depression.

	Beta (95% CI)		
	Model 1^a^^b^	Model 2^a^^c^	Model 3^a^^d^
*Aged 41–65 years* (N = 2,011)			
Underperception	0.06 (-0.33, 0.44)	0.06 (-0.33, 0.43)	0.06 (-0.32, 0.43)
Overperception	0.45* **(0.08, 0.82)**	0.42* **(0.05, 0.80)**	0.41* **(0.05, 0.78)**
DWCB		0.69* **(0.20, 1.18)**	
Fasting			0.20* **(-0.90, 1.26)**
Skipping meals			0.65* **(0.09, 1.18)**
One-food diet			1.24 (-0.01, 2.49)
Unprescribed diet pills use			0.77 (-1.13, 2.73)
*Aged 41–65 years with normal weight* (N = 1,320)			
Underperception	0.06 (-0.45, 0.55)	0.09 (-0.41, 0.57)	0.08 (-0.42, 0.58)
Overperception	0.63* **(0.22, 1.05)**	0.57* **(0.14, 0.99)**	0.57* **(0.14, 1.01)**
DWCB		1.02* **(0.32, 1.70)**	
Fasting			1.52 (-0.07, 3.10)
Skipping meals			0.57 (-0.17, 1.33)
One-food diet			1.94* **(0.23, 3.66)**
Unprescribed diet pills use			0.00 (-3.03, 2.98)
*Aged 41–65 years with obesity* (N = 691)			
Underperception	-0.34 (-0.99, 0.29)	-0.34 (-0.99, 0.29)	-0.33 (-0.98, 0.31)
DWCB		0.23 (-0.44, 0.89)	
Fasting			-0.69 (-1.97, 0.63)
Skipping meals			0.60 (-0.21, 1.42)
One-food diet			0.12 (-1.64, 1.87)
Unprescribed diet pills use			0.64 (-1.72, 3.04)

Abbreviations: PHQ-9K (Patient Health Questionnaire-9 Korean version), DWCB (disordered weight control behavior), BMI (body-mass index)

a) All models were based on four-level random intercept model, in which individuals at level 1 were nested within households at level 2, nested within neighborhoods at level 3, and nested within wider geographic areas at level 4.

b) Model 1 included weight perception pattern (accurate perception as reference), BMI (kg/m^2^), age (years), education, marital status, household income, urbanicity, severe chronic conditions, moderate-to-rigorous leisure time physical activity, smoking, and high-risk alcohol drinking.

c) Model 2 added DWCB (yes versus no) based on Model 1.

d) Model 3 added four indicator variables such as fasting (yes vs. none), skipping meals (yes vs. none), one-food diet (yes vs. none), and unprescribed diet pills use (yes vs. none), instead of overall DWCB, based on Model 1, to investigate method-specific association.

“*” indicates p-value <0.05 (two-sided).

Sensitivity analyses to address misclassification of weight perception status showed consistent directions of the associations across different cut-offs for normal weight and obesity in both women and men (Tables 6 and 7). As shown in Table 6, the magnitudes of the protective association between weight status underperception and depressive symptoms among women with obesity became more pronounced as the alternative cut-off value for BMI moved further away from 25kg/m^2^. For instance, compared with accurate perception, weight status underperception was associated with lower depressive symptoms scores by 2.32 points (0.64, 4.00) among women with BMI> = 26.0kg/m^2^, 2.73 points (0.75, 4.72) among women with BMI> = 26.5kg/m^2^, and 3.11 (0.84, 5.45) among women with BMI> = 27.0kg/m^2^. However, there was no evidence of such relationships among men with obesity.

**Table 6:** Sensitivity analysis of multilevel linear models for depressive symptoms (PHQ-9K) among Korean women with varying cut-offs for obesity.

	Women			Men		
	Model 1^a^^b^	Model 2^a^^c^	Model 3^a^^d^	Model 1^a^^b^	Model 2^a^^c^	Model 3^a^^d^
	Beta	Beta	Beta	Beta	Beta	Beta
	(95% CI)	(95% CI)	(95% CI)	(95% CI)	(95% CI)	(95% CI)
*Among those with BMI> = 26*.*0kg/m*^*2*^						
Underperception	-2.32*	-2.40*	-2.48*	-0.15	-0.17	-0.15
	**(-4.00, -0.64)**	**(-4.08, -0.72)**	**(-4.23, -0.76)**	(-1.12, 0.79)	(-1.11, 0.73)	(-1.06, 0.78)
*Among those with BMI> = 26*.*5kg/m*^*2*^						
Underperception	-2.73*	-2.76*	-2.89*	-0.19	-0.20	-0.18
	**(-4.72, -0.75)**	**(-4.76, -0.96)**	**(-4.78, -0.76)**	(-1.32, 0.97)	(-1.33, 0.91)	(-1.31, 0.97)
*Among those with BMI> = 27*.*0kg/m*^*2*^						
Underperception	-3.11*	-3.11*	-3.28*	-0.25	-0.27	-0.30
	**(-5.45, -0.84)**	**(-5.26, -0.97)**	**(-5.56, -0.92)**	(-1.71, 1.21)	(-1.74, 1.24)	(-1.80, 1.17)

Abbreviations: PHQ-9K (Patient Health Questionnaire-9 Korean version), BMI (body-mass index), DWCB (disordered weight control behavior)

a) All models were based on four-level random intercept model, in which individuals at level 1 were nested within households at level 2, nested within neighborhoods at level 3, and nested within wider geographic areas at level 4.

b) Model 1 included weight perception pattern (accurate perception as reference), BMI (kg/m^2^), age (years), menopause (for women), education, marital status, household income, urbanicity, severe chronic conditions, moderate-to-rigorous leisure time physical activity, smoking, and high-risk alcohol drinking.

c) Model 2 added DWCB (yes versus no) based on Model 1.

d) Model 3 added four indicator variables such as fasting (yes vs. none), skipping meals (yes vs. none), one-food diet (yes vs. none), and unprescribed diet pills use (yes vs. none), instead of overall DWCB, based on Model 1, to investigate method-specific association.

“*” indicates p-value <0.05 (two-sided).

**Table 7:** Sensitivity analysis of multilevel linear models for depressive symptoms (PHQ-9K) among Korean women with varying cut-offs for normal weight category.

	Women			Men		
	Model 1^a^^b^	Model 2^a^^c^	Model 3^a^^d^	Model 1^a^^b^	Model 2^a^^c^	Model 3^a^^d^
	Beta	Beta	Beta	Beta	Beta	Beta
	(95% CI)	(95% CI)	(95% CI)	(95% CI)	(95% CI)	(95% CI)
*Among those with 18*.*5< = BMI<24*.*0kg/m*^*2*^						
Underperception	0.18	0.19	0.18	0.29	0.31	0.29
	(-0.49, 0.87)	(-0.50, 0.88)	(-0.52, 0.87)	(-0.29, 0.90)	(-0.28, 0.91)	(-0.29, 0.89)
Overperception	1.05*	0.98*	0.99*	-0.34	-0.37	-0.39
	**(0.56, 1.54)**	**(0.50, 1.47)**	**(0.51, 1.48)**	(-1.26, 0.58)	(-1.32, 0.57)	(-1.31, 0.55)
*Among those with 18*.*5< = BMI<23*.*5kg/m*^*2*^						
Underperception	0.26	0.28	0.26	0.32	0.33	0.32
	(-0.46, 0.99)	(-0.43, 0.98)	(-0.45, 0.97)	(-0.27, 0.95)	(-0.30, 0.94)	(-0.31, 0.94)
Overperception	1.18*	1.12*	1.12*	-0.54	-0.51	-0.47
	**(0.66, 1.69)**	**(0.61, 1.63)**	**(0.59, 1.64)**	(-1.62, 0.57)	(-1.59, 0.60)	(-1.59, 0.63)
*Among those with 18*.*5< = BMI<23*.*0kg/m*^*2*^						
Underperception	0.35	0.36	0.34	0.41	0.43	0.42
	(-0.40, 1.09)	(-0.36, 1.10)	(-0.39, 1.10)	(-0.24, 1.07)	(-0.22, 1.07)	(-0.22, 1.08)
Overperception	1.17*	1.10*	1.08*	0.21	0.22	0.21
	**(0.60, 1.75)**	**(0.50, 1.69)**	**(0.50, 1.64)**	(-1.10, 1.52)	(-1.12, 1.57)	(-1.17, 1.57)
DWCB		0.82				
		(-0.35, 2.06)				

Abbreviations: PHQ-9K (Patient Health Questionnaire-9 Korean version), BMI (body-mass index), DWCB (disordered weight control behavior)

a) All models were based on four-level random intercept model, in which individuals at level 1 were nested within households at level 2, nested within neighborhoods at level 3, and nested within wider geographic areas at level 4.

b) Model 1 included weight perception pattern (accurate perception as reference), BMI (kg/m^2^), age (years), menopause (for women), education, marital status, household income, urbanicity, severe chronic conditions, moderate-to-rigorous leisure time physical activity, smoking, and high-risk alcohol drinking.

c) Model 2 added DWCB (yes versus no) based on Model 1.

d) Model 3 added four indicator variables such as fasting (yes vs. none), skipping meals (yes vs. none), one-food diet (yes vs. none), and unprescribed diet pills use (yes vs. none), instead of overall DWCB, based on Model 1, to investigate method-specific association.

“*” indicates p-value <0.05 (two-sided).

As presented in Table 7, weight status overperception (vs. accurate perception) was associated with higher depressive symptoms scores by 1.05 (0.56, 1.54) among women with BMI 18.5–24.0kg/m^2^, 1.18 (0.66, 1.69) among women with BMI 18.5–23.5kg/m^2^, and 1.17 (0.60, 1.75) among women with BMI 18.5–23.0kg/m^2^, suggesting a more harmful relationship among those within more apparent ranges of BMI values for normal weight status. However, such associations were not seen among men with normal weight.

Our analyses with the multiple imputation approach showed consistent results as in our primary analyses (Tables E and F in S1 File). Weight status overperception (vs. accurate perception) remained associated with higher depressive symptoms score (*β* = 0.94, 95% CI: 0.37, 1.51) among women with normal weight, whereas weight status underperception (vs. accurate perception) was associated with lower depressive symptom scores (*β* = -1.04, 95% CI: -2.01, -0.07) among women with obesity. DWCB was associated with higher depressive symptom scores (*β* = 0.80, 95% CI: 0.17, 1.43) among women. However, these relationships were not seen among men.

The multilevel binomial MCMC models with depression (PHQ-9K score of 5 or above) as the outcome showed largely similar results as in our primary analysis. As shown in Table G in S1 File, weight status overperception was associated with higher likelihood of depression among Korean women with normal weight (OR = 2.06, 95% CI: 1.48, 2.84), which is consistent with the positive association in our multilevel linear model presented in Table 2 (*β* = 1.00, 95% CI: 0.54, 1.45). Similarly, DWCB was associated with higher likelihood of depression among women (OR = 1.88, 95% CI: 1.30, 2.76), which is consistent with the positive association from the linear model shown in Table 2 (*β* = 0.88, 95% CI: 0.41, 1.35). However, there was no significant association between weight status underperception and depression among women with obesity (OR = 0.32, 95% CI: 0.07, 1.10) although the direction of the association is consistent with that from our linear model (*β* = -1.25, 95% CI: -2.44, -0.01). As in our primary analysis with linear models, there was no association between weight status perception and depression among men (Table H in S1 File).

## Discussion

Despite emerging evidence showing negative health influences of accurate weight perception among individuals with overweight/obesity, to date, little is known about the association in Asian populations. By using a nationally representative sample of Korean adults with BMI value of 18.5kg/m^2^ or above and no history of physician-diagnosed depression, we found that weight status underperception (vs. accurate perception) was cross-sectionally protectively associated with depressive symptoms in women with obesity, in which the protective association was more pronounced among those with higher BMI values. Conversely, weight status overperception (vs. accurate perception) was positively associated with depressive symptoms in women with normal weight. Moreover, disordered weight control behaviors (DWCB), particularly fasting and skipping meals, were positively associated with depressive symptoms in women.

To our knowledge, this is the first study to document a protective association between weight status underperception and depressive symptoms among women with obesity in an Asian context. Our findings converge with evidence from recent studies in Western adolescent and young adult samples [15, 16, 26]. For instance, Thurston and colleagues found that accurate weight perception among US youth with overweight/obesity with a mean age of 15.9 years was associated with increased depressive symptoms over a 12-year period [15]. Similar findings were also reported among Australian adolescents [26]. From a broader perspective, our results also align with recent research showing adverse health impacts of accurate weight perception on weight gain [7, 17] and blood pressure [9] among individuals with overweight/obesity. Together with these previous studies [7, 9, 10, 15–18, 26], our findings suggest that accurate weight perception among women with obesity may be a marker of increased risk of depression. This substantiates the emerging perspective concerning the unintended adverse consequences of the awareness-raising strategies for obesity prevention and weight management in public health and clinical practices [5–9].

To explain these associations, recent studies have pointed to the potential role of weight stigma and weight labeling [5–9]. Accumulated research has shown that individuals living with a high BMI, particularly women, often encounter weight stigma and discrimination in diverse daily-life settings such as schools, workplaces, the health care system, and in social relationships [50]. Weight stigma has been linked with adverse psychological (e.g., body dissatisfaction and depression), behavioral (e.g., disordered eating and physical inactivity), and metabolic (e.g., weight gain) outcomes [50]. Also, weight labeling (i.e., merely being told one is “too fat” by others) has also been linked with increased psychological distress and weight gain [51]. Recently, Sonneville and colleagues proposed that, although weight perception and weight labeling are different constructs, the self-perception of overweight/obese status can be viewed as “weight *self*-labeling,” which may also be associated with the process of weight stigma internalization and body dissatisfaction [7, 15, 18]. Relatedly, Unger, Kawachi, and colleagues suggested that the experience of weight discrimination and labeling might influence the self-perception of being overweight/obese, or harmful impacts of weight stigma might be stronger among those perceiving themselves as overweight/obese [9]. In line with these findings, recent studies have raised concerns that awareness-raising approaches incorporated in BMI screening and weight-loss counseling at schools and primary care practices might stigmatize individuals, thus necessitating non-stigmatizing approaches by shifting the focus from body size per se to promoting healthy behaviors and diet [5–9].

We also found that weight status overperception among women with normal weight, compared with accurate perception, was positively associated with depressive symptoms, but not among men. This replicates and extends the previous evidence, which was primarily from adolescent and young adult samples [26, 52, 53], to women in young-to-middle adulthood (aged 19–65 years). More broadly, some other studies focused on weight perception per se and presented similar findings that the perception of being overweight/obese (versus the perception of being normal weight) was positively associated with depression among US adolescents [54, 55] and female adults [25].

Prior research has suggested DWCB as the potential pathway linking weight misperception (i.e., weight overperception among individuals with normal weight and accurate weight perception among individuals with overweight/obesity) with depressive symptoms. DWCB, referring to habitual behaviors intended to control body weight (e.g., extreme food restraint, self-induced vomiting, and inappropriate use of laxatives/diuretics/diet medications), has been linked with an increased risk of depression among adolescents [55, 56]. Prior studies have also found that weight overperception among individuals with normal weight (versus accurate perception) and accurate weight perception among individuals with overweight/obesity (versus underperception) were positively associated with DWCB [18, 57]. In our analysis, although there were 4–12% changes in the magnitude of the associations between weight perception and depressive symptoms before and after adjusting for DWCB, we were not able to formally test mediational associations due to our cross-sectional design.

More importantly, our findings contribute to the current knowledge by breaking down the differential associations between specific weight control behaviors and depressive symptoms. Overall, while prior findings linking DWCB with depressive symptoms were mostly among adolescents [55, 56, 58], our findings add to the existing literature by documenting a positive association between DWCB (particularly fasting and skipping meals) and depressive symptoms among women in young-to-middle adulthood (aged 19–65 years), but not among men.

We found that one-food dieting among men with normal weight was positively associated with depressive symptoms. In South Korea, one-food dieting is amongst the most popular weight control methods. However, its approach of caloric restriction through an extremely unbalanced nutrient intake (e.g., eating only bananas for 2–3 weeks) warrants further public health investigation as the current clinical guideline for obesity management recommends the use of mild-to-moderate level caloric restriction without depriving essential nutrients through a balanced diet [33, 59].

We also found that unprescribed diet pills use was associated with higher depressive symptoms among men. Over-the-counter drugs and dietary supplements uses for weight control among both females and males has been widely documented [60]. In the Korean context, most of the anti-obesity agents including appetite suppressants, selective CB1 receptor blocker, and pancreatic lipase inhibitor are prescription drugs. However, uses of dietary supplements illegally adulterated with anti-obesity compounds has been reported among Koreans [61, 62]. Although previous studies reported associations between selective CB1 receptor blockers and psychiatric adverse events such as depressive mood disorders, anxiety, and suicidality [63–67], we were not able to obtain more detailed information on the specific medication, warranting further investigation.

This study has the following strengths. We used a nationally representative sample of non-underweight Korean adults with no history of depression and with anthropometric information objectively obtained by trained medical staff. Additionally, our models adjusted for an extensive range of variables including socio-demographic profiles, BMI (as continuous), severe chronic conditions, and behavioral variables (smoking/drinking/exercise). Moreover, to assess the robustness of our findings against the potential misclassification of weight status perception, we performed sensitivity analyses among subsamples within more restricted ranges of normal weight and obesity, which yielded consistent findings with our main results. Further sensitivity analyses with a multiple imputation approach and dichotomized outcome (depression) showed consistent results as our primary analysis. However, the following limitations should also be noted. Our cross-sectional design cannot preclude reverse causation. Additionally, small cell counts (n = 7 for normal-weight men engaged in one-food dieting) should be noted when interpreting our results. Lastly, we were not able to include other forms of disordered eating such as purging and over-/binge eating due to the limited items for DWCB in KNHANES.

## Conclusion

The findings from this study showed that weight perception and disordered weight control behaviors (DWCB) are associated with depressive symptoms in Koreans, particularly among women. Concerning the high prevalence of obesity as well as the high prevalence of weight status underperception among individuals with obesity in South Korea, recent studies have emphasized that BMI screening followed with awareness-raising approaches (i.e., correcting weight misperception) should be routinely implemented in schools, workplaces, and health-care settings [11, 13]. However, given the Korean context where the thin-ideal norm is ubiquitous, together with recent studies [5, 7, 9, 15–17], our findings raise concerns about the potential unintended consequences if the conventional BMI report card interventions are implemented without appropriate modifications to their awareness-oriented strategies. As suggested by recent studies, public health and clinical practices regarding obesity prevention and weight management may need to shift their focus away from weight per se, and move toward healthy lifestyle promotion such as through physical activity and mindful eating [5–10, 15, 16, 18].

## Supporting information

S1 File Interaction analysis for differential association across gender among Korean adults with normal weight (Table A) and obesity (Table B); Interaction analysis for differential association across age groups among Koreans with normal weight (Table C) and obesity (Table D); Sensitivity analysis by using multiply imputed datasets among women (Table E) and men (Table F); Associations of weight status perception pattern and disordered weight control behaviors with depression among Korean women (Table G) and men (Table H).
(DOCX)
