# Supplementary material for: Body weight perception, disordered weight control behaviors, and depressive symptoms among Korean adults: The Korea National Health and Nutrition Examination Survey 2014
Source: PLoS One. 2018 Jun 14;13(6):e0198841. doi: 10.1371/journal.pone.0198841 (PMC6002096; doi:10.1371/journal.pone.0198841)
Supplement: S1 File — Interaction analysis for differential association across gender among Korean adults with normal weight (Table A) and obesity (Table B); Interaction analysis for differential association across age groups among Koreans with normal weight (Table C) and obesity (Table D); Sensitivity analysis by using multiply imputed datasets among women (Table E) and men (Table F); Associations of weight status perception pattern and disordered weight control behaviors with depression among Korean women (Table G) and men (Table H). (DOCX) [file pone.0198841.s001.docx]

**Table A**.

|  | Model 1 | |  | Model 2 | |  | Model 3 | |
| --- | --- | --- | --- | --- | --- | --- | --- | --- |
|  | $\beta$ (SE) | p |  | $\beta$ (SE) | p |  | $\beta$ (SE) | p |
| Underperception | -0.16 (0.27) | 0.55 |  | -0.14 (0.27) | 0.60 |  | -0.15 (0.77) | 0.60 |
| Aged 19-40*Underperception | 0.55 (0.40) | 0.18 |  | 0.56 (0.40) | 0.17 |  | 0.54 (0.40) | 0.16 |
| Overperception | -0.01 (0.32) | 0.99 |  | -0.11 (0.33) | 0.74 |  | -0.11 (0.32) | 0.75 |
| **Aged 19-40*Overperception** | **0.77 (0.36)*** | **0.04** |  | **0.77 (0.37)*** | **0.04** |  | **0.79 (0.37)*** | **0.03** |
| DWCB |  |  |  | 1.05 (0.52) | 0.02 |  |  |  |
| Aged 19-40*DWCB |  |  |  | -0.09 (0.58) | 0.87 |  |  |  |
| Fasting |  |  |  |  |  |  | -0.30 (0.98) | 0.76 |
| **Aged 19-40*Fasting** |  |  |  |  |  |  | **3.76 (1.14)*** | **<0.01** |
| Skipping meals |  |  |  |  |  |  | 3.07 (1.27) | 0.01 |
| Aged 19-40*Skipping meals |  |  |  |  |  |  | 0.10 (0.69) | 0.89 |
| One-food diet |  |  |  |  |  |  | 10.50 (3.30) | <0.01 |
| **Aged 19-40*One-food diet** |  |  |  |  |  |  | **-2.80 (1.43)*** | **0.046** |
| Unprescribed diet pills use |  |  |  |  |  |  | 3.76 (1.14) | <0.01 |
| **Aged 19-40*Unprescribed diet pills use** |  |  |  |  |  |  | **-11.14 (3.42)*** | **<0.01** |

i) Abbreviations: PHQ-9K (Patient Health Questionnaire-9 Korean version), BMI (body-mass index), DWCB (disordered weight control behavior)

ii) All models were based on four-level random intercept model, in which individuals at level 1 were nested within households at level 2, nested within neighborhoods at level 3, and nested within wider geographic areas at level 4.

iii) Model 1 included weight perception pattern (accurate perception as reference), gender (men as reference), BMI (kg/m^2^, as a continuous variable), age (years, as a continuous variable), education (<=middle school, high school, >=college), marital status (not married, married, divorced/separated/widowed), household income (quartiles), urbanicity (rural, urban), severe chronic conditions (stroke/cancer/heart attack), moderate-to-rigorous leisure time physical activity (1=yes, 0=no), smoking (never, past, current smoker), and high-risk alcohol drinking (>=7 glasses for men, or >=5 glasses for women, of alcohol drinking episode, which persisted >=2 events/week, over the past year; 1=yes, 0=no).

iv) Model 2 added DWCB (yes versus no) based on Model 1.

v) Model 3 added four indicator variables such as fasting (yes vs. none), skipping meals (yes vs. none), one-food diet (yes vs. none), and unprescribed diet pills use (yes vs. none), instead of overall DWCB, based on Model 1, to investigate method-specific association.

vi) * indicates p-value <0.05 (two-sided), and ^ indicates p-value <0.10 (two-sided).

**Table B**.

|  | Model 1 | |  | Model 2 | |  | Model 3 | |
| --- | --- | --- | --- | --- | --- | --- | --- | --- |
|  | $\beta$ (SE) | p |  | $\beta$ (SE) | p |  | $\beta$ (SE) | p |
| Underperception | 0.17 (0.35) | 0.61 |  | 0.14 (0.34) | 0.68 |  | 0.19 (0.35) | 0.59 |
| **Aged 19-40*Underperception** | **-1.57 (0.65)*** | **0.02** |  | **-1.55 (0.66)*** | **0.02** |  | **-1.70 (0.65)*** | **0.01** |
| DWCB |  |  |  | -0.17 (0.38) | 0.65 |  |  |  |
| **Aged 19-40*DWCB** |  |  |  | **1.05 (0.54)^** | **0.052** |  |  |  |
| Fasting |  |  |  |  |  |  | -0.74 (0.68) | 0.27 |
| Aged 19-40*Fasting |  |  |  |  |  |  | 0.29 (1.00) | 0.77 |
| Skipping meals |  |  |  |  |  |  | 0.06 (0.43) | 0.89 |
| **Aged 19-40*Skipping meals** |  |  |  |  |  |  | **1.67 (0.62)*** | **0.01** |
| One-food diet |  |  |  |  |  |  | -1.05 (1.71) | 0.54 |
| Aged 19-40*One-food diet |  |  |  |  |  |  | 1.22 (1.93) | 0.52 |
| Unprescribed diet pills use |  |  |  |  |  |  | 5.01 (3.02) | 0.10 |
| **Aged 19-40*Unprescribed diet pills use** |  |  |  |  |  |  | **-6.14 (3.17)^** | **0.052** |

i) Abbreviations: PHQ-9K (Patient Health Questionnaire-9 Korean version), BMI (body-mass index), DWCB (disordered weight control behavior)

ii) All models were based on four-level random intercept model, in which individuals at level 1 were nested within households at level 2, nested within neighborhoods at level 3, and nested within wider geographic areas at level 4.

iii) Model 1 included weight perception pattern (accurate perception as reference), gender (men as reference), BMI (kg/m^2^, as a continuous variable), age (years, as a continuous variable), education (<=middle school, high school, >=college), marital status (not married, married, divorced/separated/widowed), household income (quartiles), urbanicity (rural, urban), severe chronic conditions (stroke/cancer/heart attack), moderate-to-rigorous leisure time physical activity (1=yes, 0=no), smoking (never, past, current smoker), and high-risk alcohol drinking (>=7 glasses for men, or >=5 glasses for women, of alcohol drinking episode, which persisted >=2 events/week, over the past year; 1=yes, 0=no).

iv) Model 2 added DWCB (yes versus no) based on Model 1.

v) Model 3 added four indicator variables such as fasting (yes vs. none), skipping meals (yes vs. none), one-food diet (yes vs. none), and unprescribed diet pills use (yes vs. none), instead of overall DWCB, based on Model 1, to investigate method-specific association.

vi) * indicates p-value <0.05 (two-sided), and ^ indicates p-value <0.10 (two-sided).

**Table C**.

|  | Model 1 | |  | Model 2 | |  | Model 3 | |
| --- | --- | --- | --- | --- | --- | --- | --- | --- |
|  | $\beta$ (SE) | p |  | $\beta$ (SE) | p |  | $\beta$ (SE) | p |
| Underperception | -0.21 (0.25) | 0.43 |  | -0.17 (0.25) | 0.48 |  | -0.19 (0.25) | 0.45 |
| Aged 19-40*Underperception | -0.09 (0.38) | 0.80 |  | 0.00 (0.38) | 0.99 |  | -0.03 (0.38) | 0.93 |
| Overperception | **0.66* (0.21)** | **0.00** |  | **0.59* (0.22)** | **0.01** |  | **0.60 (0.21)*** | **0.00** |
| Aged 19-40*Overperception | 0.34 (0.29) | 0.23 |  | 0.21 (0.29) | 0.46 |  | 0.21 (0.29) | 0.46 |
| DWCB |  |  |  | **0.91* (0.35)** | **0.01** |  |  |  |
| Aged 19-40*DWCB |  |  |  | 0.30 (0.46) | 0.52 |  |  |  |
| Fasting |  |  |  |  |  |  | 1.46 (0.85)^ | 0.08 |
| Aged 19-40*Fasting |  |  |  |  |  |  | 1.72 (1.07) | 0.11 |
| Skipping meals |  |  |  |  |  |  | 0.53 (0.40) | 0.18 |
| Aged 19-40*Skipping meals |  |  |  |  |  |  | -0.10 (0.53) | 0.85 |
| One-food diet |  |  |  |  |  |  | **1.83 (0.93)*** | **0.046** |
| Aged 19-40*One-food diet |  |  |  |  |  |  | -1.54 (1.20) | 0.21 |
| Unprescribed diet pills use |  |  |  |  |  |  | -0.21 (1.65) | 0.89 |
| Aged 19-40*Unprescribed diet pills use |  |  |  |  |  |  | 0.64 (2.01) | 0.75 |

i) Abbreviations: PHQ-9K (Patient Health Questionnaire-9 Korean version), BMI (body-mass index), DWCB (disordered weight control behavior)

ii) All models were based on four-level random intercept model, in which individuals at level 1 were nested within households at level 2, nested within neighborhoods at level 3, and nested within wider geographic areas at level 4.

iii) Model 1 included weight perception pattern (accurate perception as reference), gender (men as reference), BMI (kg/m^2^, as a continuous variable), age (years, as a continuous variable), education (<=middle school, high school, >=college), marital status (not married, married, divorced/separated/widowed), household income (quartiles), urbanicity (rural, urban), severe chronic conditions (stroke/cancer/heart attack), moderate-to-rigorous leisure time physical activity (1=yes, 0=no), smoking (never, past, current smoker), and high-risk alcohol drinking (>=7 glasses for men, or >=5 glasses for women, of alcohol drinking episode, which persisted >=2 events/week, over the past year; 1=yes, 0=no).

iv) Model 2 added DWCB (yes versus no) based on Model 1.

v) Model 3 added four indicator variables such as fasting (yes vs. none), skipping meals (yes vs. none), one-food diet (yes vs. none), and unprescribed diet pills use (yes vs. none), instead of overall DWCB, based on Model 1, to investigate method-specific association.

vi) * indicates p-value <0.05 (two-sided), and ^ indicates p-value <0.10 (two-sided).

**Table D**.

|  | Model 1 | |  | Model 2 | |  | Model 3 | |
| --- | --- | --- | --- | --- | --- | --- | --- | --- |
|  | $\beta$ (SE) | p |  | $\beta$ (SE) | p |  | $\beta$ (SE) | p |
| Underperception | -0.33 (0.33) | 0.32 |  | -0.32 (0.33) | 0.34 |  | -0.31 (0.34) | 0.36 |
| Aged 19-40*Underperception | -0.30 (0.68) | 0.67 |  | -0.26 (0.71) | 0.72 |  | -0.27 (0.70) | 0.69 |
| DWCB |  |  |  | **0.07* (0.36)** | **0.01** |  |  |  |
| Aged 19-40*DWCB |  |  |  | 0.66 (0.53) | 0.52 |  |  |  |
| Fasting |  |  |  |  |  |  | -0.79 (0.70) | 0.27 |
| Aged 19-40*Fasting |  |  |  |  |  |  | 0.61 (1.00) | 0.55 |
| Skipping meals |  |  |  |  |  |  | 0.48 (0.43) | 0.26 |
| Aged 19-40*Skipping meals |  |  |  |  |  |  | 0.65 (0.63) | 0.30 |
| One-food diet |  |  |  |  |  |  | 0.21 (0.91) | 0.84 |
| Aged 19-40*One-food diet |  |  |  |  |  |  | -0.01 (1.81) | 0.98 |
| Unprescribed diet pills use |  |  |  |  |  |  | 0.18 (1.26) | 0.88 |
| Aged 19-40*Unprescribed diet pills use |  |  |  |  |  |  | -0.66 (1.99) | 0.73 |

i) Abbreviations: PHQ-9K (Patient Health Questionnaire-9 Korean version), BMI (body-mass index), DWCB (disordered weight control behavior)

ii) All models were based on four-level random intercept model, in which individuals at level 1 were nested within households at level 2, nested within neighborhoods at level 3, and nested within wider geographic areas at level 4.

iii) Model 1 included weight perception pattern (accurate perception as reference), gender (men as reference), BMI (kg/m^2^, as a continuous variable), age (years, as a continuous variable), education (<=middle school, high school, >=college), marital status (not married, married, divorced/separated/widowed), household income (quartiles), urbanicity (rural, urban), severe chronic conditions (stroke/cancer/heart attack), moderate-to-rigorous leisure time physical activity (1=yes, 0=no), smoking (never, past, current smoker), and high-risk alcohol drinking (>=7 glasses for men, or >=5 glasses for women, of alcohol drinking episode, which persisted >=2 events/week, over the past year; 1=yes, 0=no).

iv) Model 2 added DWCB (yes versus no) based on Model 1.

v) Model 3 added four indicator variables such as fasting (yes vs. none), skipping meals (yes vs. none), one-food diet (yes vs. none), and unprescribed diet pills use (yes vs. none), instead of overall DWCB, based on Model 1, to investigate method-specific association.

vi) * indicates p-value <0.05 (two-sided), and ^ indicates p-value <0.10 (two-sided).

**Table E**.

|  | All women participants  (BMI>=18.5kg/m^2^) | |  | Women with normal weight (18.5<=BMI<25.0kg/m^2^) | |  | Women with obesity (BMI>=25.0kg/m^2^) | |
| --- | --- | --- | --- | --- | --- | --- | --- | --- |
|  | Model 1  Beta  (95% CI) | Model 2  Beta  (95% CI) |  | Model 1  Beta  (95% CI) | Model 2  Beta  (95% CI) |  | Model 1  Beta  (95% CI) | Model 2  Beta  (95% CI) |
| Underperception | -0.01  (-0.63, 0.61) | 0.02  (-0.59, 0.64) |  | -0.14  (-0.92, 0.63) | -0.10  (-0.88, 0.68) |  | **-1.04***  **(-2.01, -0.07)** | **-1.03***  **(-2.01, -0.04)** |
| Overperception | **0.45***  **(0.01, 0.89)** | 0.40  (-0.04, 0.85) |  | **0.94***  **(0.37, 1.51)** | **0.87***  **(0.30, 1.45)** |  |  |  |
| DWCB |  | **0.80***  **(0.17, 1.43)** |  |  | 0.70  (-0.07, 1.46) |  |  | 0.90  (-0.09, 1.90) |

i) Abbreviations: PHQ-9K (Patient Health Questionnaire-9 Korean version), BMI (body-mass index), DWCB (disordered weight control behavior)

ii) Multiple imputation was performed by using the chained equations method implemented in Stata/MP 15.0 with the option of 20 imputed datasets and 100 iterations for the burn-in period.

iii) All models adjusted for complex survey design and covariates such as BMI (kg/m^2^, as a continuous variable), age (years, as a continuous variable), menopause, education (<=middle school, high school, >=college), marital status (not married, married, divorced/separated/widowed), household income (quartiles), urbanicity (rural, urban), severe chronic conditions (stroke/cancer/heart attack), moderate-to-rigorous leisure time physical activity (1=yes, 0=no), smoking (never, past, current smoker), and high-risk alcohol drinking (>=7 glasses for men, or >=5 glasses for women, of alcohol drinking episode, which persisted >=2 events/week, over the past year; 1=yes, 0=no).

vi) * indicates p-value <0.05 (two-sided).

**Table F**.

|  | All men participants  (BMI>=18.5kg/m^2^) | |  | Men with normal weight (18.5<=BMI<25.0kg/m^2^) | |  | Men with obesity  (BMI>=25.0kg/m^2^) | |
| --- | --- | --- | --- | --- | --- | --- | --- | --- |
|  | Model 1  Beta  (95% CI) | Model 2  Beta  (95% CI) |  | Model 1  Beta  (95% CI) | Model 2  Beta  (95% CI) |  | Model 1  Beta  (95% CI) | Model 2  Beta  (95% CI) |
| Underperception | 0.18  (-0.29, 0.65) | 0.19  (-0.29, 0.67) |  | 0.34  (-0.30, 0.98) | 0.35  (-0.30, 0.99) |  | 0.13  (-0.74, 1.01) | 0.13  (-0.75, 1.01) |
| Overperception | -0.02  (-0.66, 0.61) | -0.05  (-0.68, 0.58) |  | -0.13  (-0.79, 0.53) | -0.21  (-0.85, 0.44) |  |  |  |
| DWCB |  | 0.36  (-0.35, 1.06) |  |  | 0.97  (-0.12, 2.07) |  |  | -0.04  (-0.93, 0.85) |

i) Abbreviations: PHQ-9K (Patient Health Questionnaire-9 Korean version), BMI (body-mass index), DWCB (disordered weight control behavior)

ii) Multiple imputation was performed by using the chained equations method implemented in Stata/MP 15.0 with the option of 20 imputed datasets and 100 iterations for the burn-in period.

iii) All models adjusted for complex survey design and covariates such as BMI (kg/m^2^, as a continuous variable), age (years, as a continuous variable), menopause, education (<=middle school, high school, >=college), marital status (not married, married, divorced/separated/widowed), household income (quartiles), urbanicity (rural, urban), severe chronic conditions (stroke/cancer/heart attack), moderate-to-rigorous leisure time physical activity (1=yes, 0=no), smoking (never, past, current smoker), and high-risk alcohol drinking (>=7 glasses for men, or >=5 glasses for women, of alcohol drinking episode, which persisted >=2 events/week, over the past year; 1=yes, 0=no).

vi) * indicates p-value <0.05 (two-sided).

**Table G.**

|  | OR (95% CI) | | |
| --- | --- | --- | --- |
|  | Model 1^ab^ | Model 2^ac^ | Model 3^ad^ |
| *All Women* (N=1,876) |  |  |  |
| Underperception | 1.33 (0.75, 2.36) | 1.23 (0.73, 2.12) | 1.27 (0.76, 2.04) |
| Overperception | 1.42 (1.00, 2.06) | 1.29 (0.94, 1.75) | 1.27 (0.97, 1.64) |
| DWCB |  | **1.88* (1.30, 2.76)** |  |
| Fasting |  |  | 1.64 (0.81, 3.28) |
| Skipping meals |  |  | **1.66* (1.12, 2.46)** |
| One-food diet |  |  | 1.17 (0.53, 2.70) |
| Unprescribed diet pills use |  |  | 0.93 (0.29, 2.84) |
| *Women with Normal Weight* (N=1,410) | | | |
| Underperception | 1.11 (0.68, 1.80) | 1.11 (0.58, 2.04) | 1.03 (0.61, 1.69) |
| Overperception | **2.06* (1.48, 2.84)** | **2.30* (1.44, 4.25)** | **2.25* (1.62, 3.22)** |
| DWCB |  | **1.88* (1.19, 3.40)** |  |
| Fasting |  |  | 2.44 (0.96, 5.88) |
| Skipping meals |  |  | 1.36 (0.84, 2.12) |
| One-food diet |  |  | 1.19 (0.44, 2.97) |
| Unprescribed diet pills use |  |  | 0.56 (0.10, 2.64) |
| *Women with Obesity* (N=466) | | | |
| Underperception | 0.32 (0.07, 1.10) | **0.00* (0.00, 0.17)** | 0.27 (0.05, 1.08) |
|  |  |  |  |
| DWCB |  | **4.5×10^19^* (1.11, 10.2×10^42^)** |  |
| Fasting |  |  | 0.96 (0.23, 3.55) |
| Skipping meals |  |  | **3.55* (1.48, 8.85)** |
| One-food diet |  |  | 0.72 (0.11, 4.38) |
| Unprescribed diet pills use |  |  | 0.66 (0.09, 4.08) |

Abbreviations: PHQ-9K (Patient Health Questionnaire-9 Korean version), DWCB (disordered weight control behavior), BMI (body-mass index)

a) All models were based on four-level random intercept model, in which individuals at level 1 were nested within households at level 2, nested within neighborhoods at level 3, and nested within wider geographic areas at level 4.

b) Model 1 included weight perception pattern (accurate perception as reference), BMI (kg/m^2^), age (years), menopause (for women), education, marital status, household income, urbanicity, severe chronic conditions, moderate-to-rigorous leisure time physical activity, smoking, and high-risk alcohol drinking.

c) Model 2 added DWCB (yes versus no) based on Model 1.

d) Model 3 added four indicator variables such as fasting (yes vs. none), skipping meals (yes vs. none), one-food diet (yes vs. none), and unprescribed diet pills use (yes vs. none), instead of overall DWCB, based on Model 1, to investigate method-specific association.

“*” indicates p-value <0.05 (two-sided).

**Table H.**

|  | OR (95% CI) | | |
| --- | --- | --- | --- |
|  | Model 1 | Model 2 | Model 3 |
| *All Men* (N=1,442) |  |  |  |
| Underperception | 0.90 (0.58, 1.39) | 0.91 (0.60, 1.35) | 1.32 (0.67, 4.11) |
| Overperception | 0.75 (0.41, 1.36) | 0.74 (0.40, 1.26) | 0.75 (0.23, 2.32) |
| DWCB |  | 1.31 (0.75, 2.21) |  |
| Fasting |  |  | 0.13 (0.00, 1.36) |
| Skipping meals |  |  | 2.14 (0.68, 8.31) |
| One-food diet |  |  | 11.29 (0.64, 603.05) |
|  |  |  |  |
| *Men with Normal Weight* (N=852) | | | |
| Underperception | 0.86 (0.54, 1.38) | 1.01 (0.62, 1.59) | 1.16 (0.70, 1.90) |
| Overperception | 0.70 (0.37, 1.29) | 0.63 (0.33, 1.13) | 0.60 (0.32, 1.12) |
| DWCB |  | 1.73 (0.74, 4.01) |  |
| Fasting |  |  | 0.53 (0.06, 3.07) |
| Skipping meals |  |  | 1.50 (0.46, 4.15) |
| One-food diet |  |  | 4.23 (0.64, 25.26) |
|  |  |  |  |
| *Men with Obesity* (N=590) | | | |
| Underperception | 0.76 (0.12, 2.73) | 0.01 (0.00, 3.3**×**10^21^) | 0.66 (0.00, 1.4**×**10^12^) |
|  |  |  |  |

Abbreviations: PHQ-9K (Patient Health Questionnaire-9 Korean version), DWCB (disordered weight control behavior), BMI (body-mass index)

a) All models were based on four-level random intercept model, in which individuals at level 1 were nested within households at level 2, nested within neighborhoods at level 3, and nested within wider geographic areas at level 4.

b) Model 1 included weight perception pattern (accurate perception as reference), BMI (kg/m^2^), age (years), education, marital status, household income, urbanicity, severe chronic conditions, moderate-to-rigorous leisure time physical activity, smoking, and high-risk alcohol drinking.

c) Model 2 added DWCB (yes versus no) based on Model 1.

d) Model 3 added four indicator variables such as fasting (yes vs. none), skipping meals (yes vs. none), one-food diet (yes vs. none), and unprescribed diet pills use (yes vs. none), instead of overall DWCB, based on Model 1, to investigate method-specific association.

e) Among men with obesity, coefficients for DWCB and its each component were not estimated.

“*” indicates p-value <0.05 (two-sided).
